# Deubiquitinases as Regulators and Therapeutic Targets in Vascular Diseases

**DOI:** 10.3390/ijms27188350

**Published:** 2026-09-19

**Authors:** Zi-Kun Lin, Lei Wang

**Affiliations:** 1 Department of Nutrition and Food Hygiene, Beijing Key Laboratory of Environment and Aging, School of Public Health, Capital Medical University, Beijing 100069, China; 1491902983@qq.com; 2 The Key Laboratory of Remodeling-Related Cardiovascular Disease, Ministry of Education, Beijing 100029, China; 3 School of Basic Medical Sciences, Capital Medical University, Beijing 100069, China

**Keywords:** deubiquitinases (DUBs), vascular diseases, vascular remodeling, vascular inflammation, angiogenesis, therapeutic targets

## Abstract

Ubiquitination and deubiquitination are dynamic post-translational regulatory processes that control protein stability and signaling. Deubiquitinases (DUBs), which remove ubiquitin chains from target proteins, have emerged as important regulators of vascular homeostasis and disease. In this review, we summarize recent progress in defining the roles of DUBs in major vascular pathologies, including vascular inflammation, remodeling, neointimal formation, angiogenesis, endothelial barrier dysfunction, thrombosis, and fibrosis. Accumulating evidence indicates that distinct DUBs act in a cell type- and context-dependent manner in endothelial cells, vascular smooth muscle cells, immune cells, and fibroblasts, contributing to vascular diseases such as aortic aneurysm, atherosclerosis, pulmonary hypertension, ischemic stroke, and intimal hyperplasia. These findings support DUBs as key regulatory nodes integrating ubiquitin signaling with inflammatory and stress-response pathways in the vasculature. In parallel, efforts to pharmacologically modulate DUB activity have led to the development of selective inhibitors and activators, although challenges remain in achieving substrate specificity and tissue selectivity. Overall, DUBs represent promising therapeutic targets for vascular disease, and future studies should focus on defining disease-specific DUB–substrate networks and developing context-dependent strategies for precise vascular intervention.

## 1. Introduction

Protein homeostasis in eukaryotic cells is maintained through a delicate balance between protein synthesis and degradation. Ubiquitination involves the covalent attachment of ubiquitin to substrate proteins, marking them for proteasomal degradation (via K48-linked chains) or modulating their function (e.g., K63-linked chains in signaling). This process is mediated by a sequential enzymatic cascade comprising ubiquitin-activating (E1), conjugating (E2), and ligating (E3) enzymes [1,2]. Deubiquitination, mediated by ~100 human deubiquitinases (DUBs), reverses ubiquitination and fine-tunes protein stability and signaling essential for cellular homeostasis [3]. Perturbation of the ubiquitination–deubiquitination balance disrupts vascular cell function, including endothelial integrity, smooth muscle cell phenotype, inflammation, and extracellular matrix remodeling. Consequently, DUBs have emerged as central regulators of vascular pathophysiology and promising therapeutic targets. This review systematically summarizes DUB families, delineates their disease- and cell type-specific roles in major vascular pathologies, and discusses their emerging therapeutic potential.

This review was based on a targeted literature search of the PubMed and Web of Science databases. Search terms included deubiquitinase families (“deubiquitinase”, “deubiquitylase”, “ubiquitin-specific protease”, “USP”, “OTU domain”, “UCH”), vascular biology and disease (“vascular”, “endothelial”, “vascular smooth muscle”, “atherosclerosis”, “aortic aneurysm”, “pulmonary hypertension”, “ischemic stroke”, “thrombosis”, “intimal hyperplasia”, “angiogenesis”, “vascular remodeling”), and therapeutic modulation (“DUB inhibitor”, “ubiquitin-activating enzyme”, “gene therapy”). Titles and abstracts were screened, and full texts were assessed for relevance. Original research and review articles published in peer-reviewed journals that provided direct evidence regarding DUB expression, substrate-level regulation, or therapeutic targeting in vascular cells or vascular disease models were included. Reference lists of eligible publications were manually screened to identify additional relevant studies. Priority was given to publications from the past five years, as well as to studies providing genetic loss- or gain-of-function evidence or pharmacological validation. In total, 83 publications met the inclusion criteria and were cited in this review.

## 2. Molecular Mechanisms of Deubiquitinases in Vascular Pathophysiology

### 2.1. Regulation of Vascular Inflammatory Signaling

Deubiquitinases (DUBs) precisely regulate vascular inflammatory response by modulating ubiquitination of key components within the nuclear factor-κB (NF-κB), mitogen-activated protein kinase (MAPK), and NLRP3 inflammasome pathways. Through substrate- and linkage-specific deubiquitination, DUBs control the amplitude, duration, and termination of inflammatory signaling in endothelial cells, vascular smooth muscle cells (VSMCs), and immune cells. Several DUBs restrain NF-κB activation by removing activating ubiquitin chains from proximal signaling components. USP20, USP25, CYLD, Cezanne (OTUD7B), OTUD1, OTULIN, and A20 (TNFAIP3) regulate signaling complexes containing TNF receptor-associated factor 6 (TRAF6), receptor-interacting protein kinase 1 (RIPK1), NF-κB essential modulator (NEMO/IKKγ), or related adaptor proteins. USP20 removes K63-linked ubiquitin chains from TRAF6 and modulates β-arrestin-dependent inflammatory signaling. USP25 deubiquitinates TAB2 and TRAF family proteins, thereby limiting downstream TAK1/IKK activation. CYLD removes primarily K63-linked ubiquitin chains from TRAF2, TRAF6, NEMO, and RIPK1, thus restraining NF-κB signaling and influencing cell-death decisions. OTUD1 deubiquitinates RIP2 to suppress inflammatory signaling, whereas OTULIN selectively disassembles Met1-linked linear ubiquitin chains generated by the linear ubiquitin chain assembly complex (LUBAC). In addition, A20, an NF-κB-inducible DUB, establishes a negative-feedback loop that prevents excessive or sustained inflammatory activation [3,4,5,6,7,8,9,10]. These substrates function as key hubs in inflammatory signal transduction. TRAF6 acts as an E3 ubiquitin ligase that facilitates activation of the IKK complex and MAPK cascades; RIPK1 coordinates cell-survival, inflammatory, and cell-death signaling; NEMO is the essential regulatory subunit of the IKK complex; and TAK1 integrates upstream ubiquitin-dependent signals to activate IKK and downstream MAPKs. DUB-mediated editing of ubiquitin chains at these nodes therefore provides a critical checkpoint for inflammatory homeostasis.

DUBs also regulate MAPK activation and NLRP3 inflammasome signaling [11]. USP29 suppresses MAPK signaling through deubiquitination of TAK1, while CYLD limits ERK and p38 activation [12,13]. In the NLRP3 inflammasome pathway, DUBs display bidirectional effects: USP5, USP22, and CYLD facilitate autophagic degradation of NLRP3, thereby limiting inflammasome activation. Conversely, OTUD6A, UCH-L1, and BRCC3 remove K48-linked ubiquitin chains from NLRP3, stabilize the inflammasome sensor, and promote NLRP3 oligomerization, ASC speck formation, caspase-1 activation, and interleukin-1β maturation [3,6,14,15,16,17]. Additionally, USP9X limits oxidized LDL uptake by deubiquitinating SR-A1, while OTUD6A exacerbates inflammation via stabilization of NLRP3 inflammasome components [5,16,18]. These opposing actions illustrate that NLRP3 activity is determined by the balance between DUBs that promote its degradation and those that prevent its proteasomal turnover.

**Figure 1:**
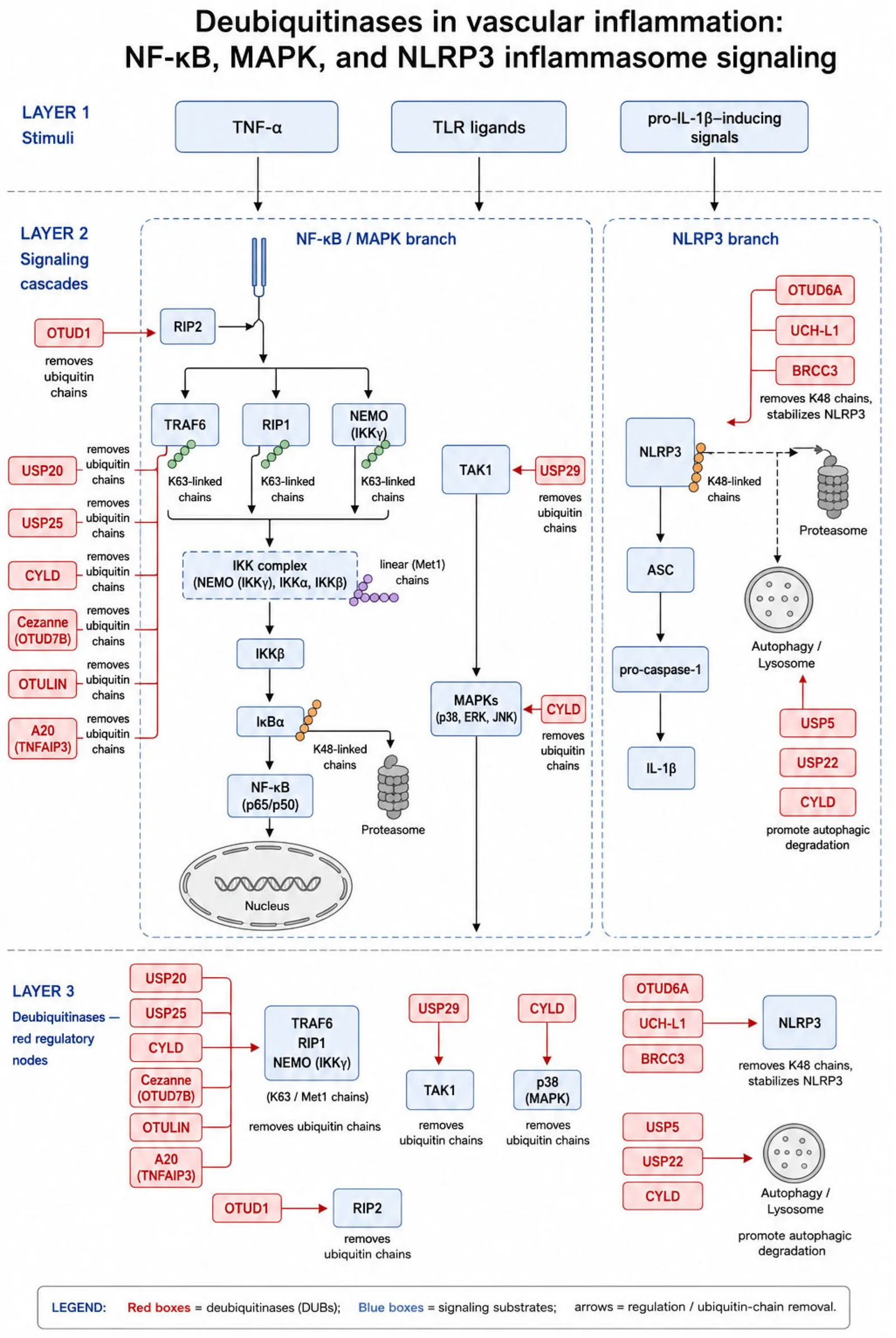
**Schematic overview of key deubiquitinases and proximal signaling molecules involved in inflammatory signaling.** K63- and Met1-linked ubiquitin chains assembled on TRAF6, RIP1, and NEMO activate the IKK complex and downstream MAPK/NF-κB signaling. USP20, USP25, CYLD, Cezanne (OTUD7B), OTULIN, and A20 (TNFAIP3) remove these chains to restrain signaling; USP29 targets TAK1, and OTUD1 targets RIP2. In the NLRP3 inflammasome branch, OTUD6A, UCH-L1, and BRCC3 remove K48-linked chains from NLRP3 to stabilize it and promote inflammasome activation and IL-1β maturation, whereas USP5, USP22, and CYLD promote autophagic degradation of NLRP3. DUBs are shown in red, signaling substrates in blue; arrows indicate regulation and ubiquitin-chain removal.

Moreover, DUBs regulate inflammatory cell-death pathways. CYLD can promote necroptotic signaling by deubiquitinating RIPK1 and RIPK3 and facilitating necrosome assembly, whereas OTUB1 and OTULIN suppress necroptosis by maintaining pro-survival signaling complexes [19]. DUBs also influence apoptosis, mitochondrial quality control, and innate immune activation. For example, OTUD1 stabilizes PINK1 and promotes mitophagy, whereas USP11 contributes to STING-dependent inflammatory activation through the OTUD5–STING axis [20,21]. Collectively, these findings establish DUBs as key modulators of inflammatory signaling and inflammatory cell death in vascular cells (Figure 1).

### 2.2. Regulation of Vascular Cell Phenotype, Proliferation, and Fibrotic Signaling

DUBs regulate vascular remodeling-related cellular processes by controlling vascular smooth muscle cell (VSMC) phenotypic plasticity, cell-cycle progression, growth-factor signaling, extracellular matrix homeostasis, and fibrotic responses. In particular, ubiquitin-dependent regulation of key proliferative, migratory, and profibrotic proteins determines the balance between vascular repair and pathological remodeling. The transition of VSMCs from a contractile to a synthetic phenotype requires downregulation of contractile markers, including SM22α and α-smooth muscle actin (α-SMA), together with cell-cycle re-entry. USP10 and USP14 promote this process by stabilizing Skp2, an F-box component of the SKP1–CUL1–F-box (SCF) ubiquitin ligase complex [22,23]. Stabilized Skp2 enhances degradation of the cyclin-dependent kinase inhibitor p27, thereby facilitating G1/S-phase progression and VSMC proliferation. OTUB1 promotes proliferative and migratory signaling by preventing K48-linked ubiquitination and degradation of platelet-derived growth factor receptor β (PDGFRβ), thereby sustaining PDGF-BB-induced downstream signaling [24]. CYLD can also modulate VSMC behavior through its effects on ERK/p38 signaling [22,23,24,25]. In contrast, JOSD2 restrains VSMC proliferation and migration by stabilizing SMAD7, an endogenous inhibitory regulator of transforming growth factor-β (TGF-β) signaling [26]. Thus, DUB-dependent regulation of Skp2, PDGFRβ, and SMAD7 represents a central molecular mechanism through which ubiquitin signaling controls VSMC phenotypic switching and proliferative capacity.

**Figure 2:**
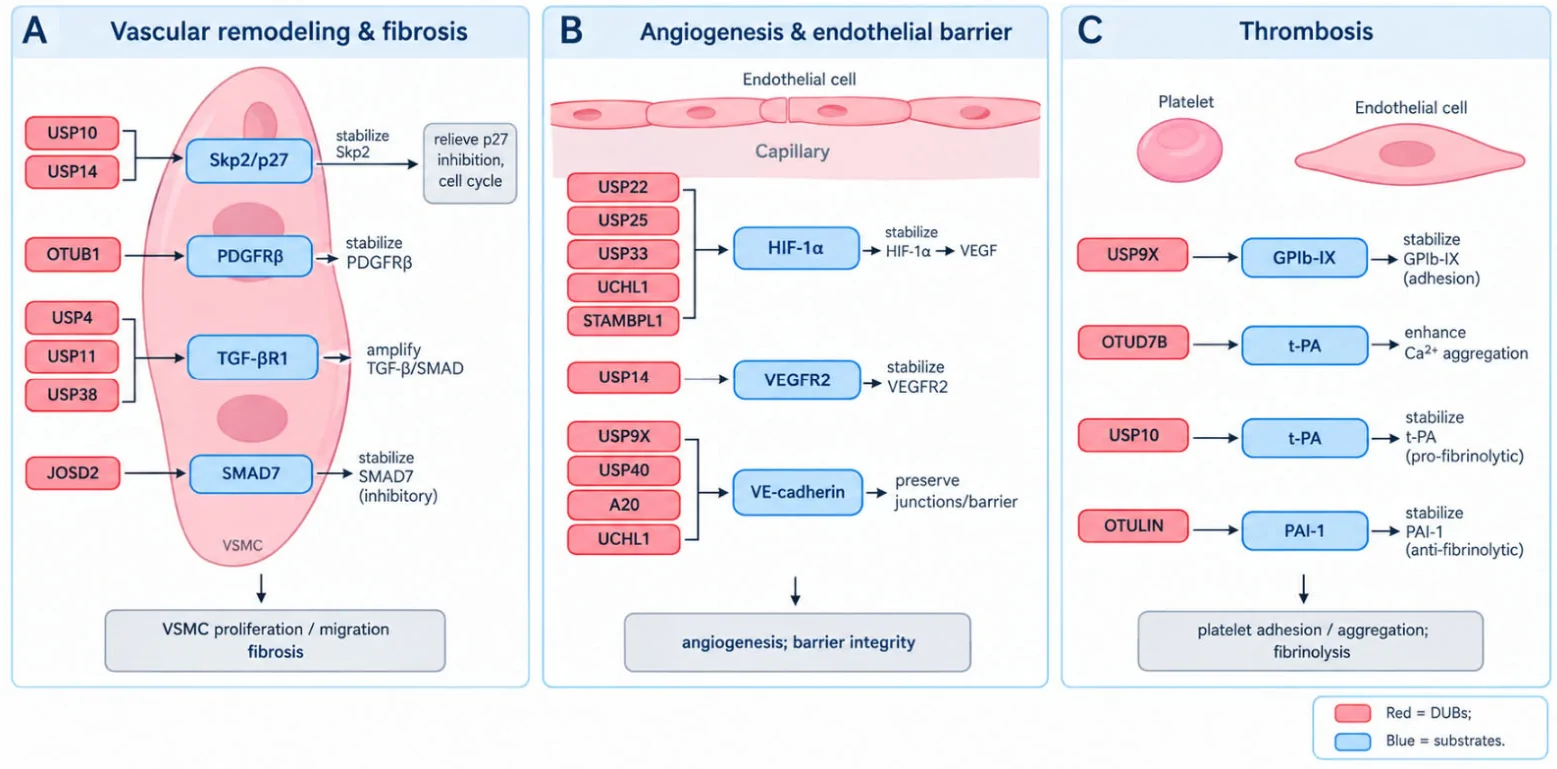
**Deubiquitinase regulation of vascular cell phenotype, angiogenesis, endothelial barrier integrity, and hemostatic pathways.** (**A**) USP10 and USP14 stabilize Skp2 to relieve p27-mediated cell-cycle inhibition; OTUB1 stabilizes PDGFRβ; USP4, USP11, and USP38 amplify TGF-β/SMAD signaling to drive fibrosis, whereas JOSD2 stabilizes the inhibitor SMAD7. (**B**) USP22, USP25, USP33, UCHL1, and STAMBPL1 stabilize HIF-1α to sustain VEGF signaling; USP14 stabilizes VEGFR2; USP9X, USP40, A20, and UCHL1 preserve endothelial barrier integrity. (**C**) USP9X stabilizes the platelet GPIb-IX complex and OTUD7B enhances Ca^2+^-dependent aggregation, while USP10 (t-PA) and OTULIN (PAI-1) reciprocally regulate fibrinolysis. DUBs are shown in red and substrates in blue.

DUBs regulate fibrotic signaling and extracellular matrix remodeling. USP4, USP11, and USP38 promote profibrotic responses by stabilizing TGF-β receptor type I (TGF-βR1), epidermal growth factor receptor (EGFR), and the TGF-β signaling scaffold STRAP, respectively [27,28,29,30]. Stabilization of TGF-βR1 prolongs SMAD2/3 phosphorylation and enhances profibrotic gene expression, whereas USP11- and USP38-dependent regulation of EGFR and STRAP further amplifies TGF-β/SMAD signaling. These mechanisms support fibroblast activation, endothelial-to-mesenchymal transition, and extracellular matrix deposition. Conversely, USP15 may mitigate fibrotic responses by enhancing matrix metalloproteinase expression and facilitating extracellular matrix turnover [31]. Moreover, USP36 stabilizes superoxide dismutase 2, thereby reducing reactive oxygen species accumulation and limiting oxidative stress, a major driver of cellular dysfunction and fibrotic signaling [32]. Together, these findings indicate that DUBs govern vascular-cell plasticity and remodeling through coordinated regulation of cell-cycle checkpoints, receptor turnover, TGF-β/SMAD signaling, extracellular matrix dynamics, and redox homeostasis (Figure 2).

### 2.3. Regulation of Angiogenesis, Endothelial Barrier Integrity, Platelet Function and Fibrinolysis

DUBs regulate angiogenesis by controlling the stability and activity of central proangiogenic factors, including hypoxia-inducible factor 1α (HIF-1α), vascular endothelial growth factor receptor 2 (VEGFR2), β-catenin, and Notch1. USP22, USP25, USP33, UCHL1, and STAMBPL1 promote HIF-1α stability and thereby enhance vascular endothelial growth factor (VEGF)-dependent angiogenic signaling [33,34,35,36,37,38,39]. Under normoxic conditions, HIF-1α is hydroxylated by prolyl hydroxylases and subsequently targeted for von Hippel–Lindau (VHL)-dependent K48-linked ubiquitination and proteasomal degradation. DUB-mediated removal of degradative ubiquitin chains extends the half-life of HIF-1α and sustains VEGF transcription. DUBs also regulate signaling events downstream of VEGF. USP14 stabilizes VEGFR2 and prolongs VEGF-induced endothelial-cell proliferation, migration, and tube formation. In parallel, USP9X-mediated stabilization of β-catenin and USP7-dependent suppression of Notch1 contribute to the regulation of endothelial-cell specification and vascular sprouting [33,34,35,36,37,38,39]. Through these interconnected mechanisms, DUBs influence both the initiation and maturation of angiogenic responses.

Beyond angiogenesis, DUBs are essential for the maintenance of endothelial barrier integrity. USP9X, USP40, A20, and UCHL1 preserve endothelial homeostasis by regulating adherens junctions, tight junctions, cytoskeletal organization, and inflammation-associated permeability changes [40,41,42,43,44,45,46]. USP9X supports Wnt/β-catenin signaling required for endothelial barrier formation and maintenance, whereas USP40 stabilizes HSP90β and protects endothelial integrity. A20 restricts inflammatory signaling that would otherwise disrupt intercellular junctions, while UCHL1 modulates endothelial permeability. Loss or dysregulation of these protective mechanisms may result in junctional disassembly, increased vascular permeability, edema, and tissue injury. Therefore, DUB-mediated regulation of angiogenic and barrier-related signaling is critical for vascular homeostasis (Figure 2).

DUBs contribute to hemostatic balance by regulating platelet adhesion, platelet activation, and fibrinolytic activity. In platelets, USP9X stabilizes the glycoprotein Ib–IX (GPIb-IX) complex, which is required for platelet adhesion under high-shear conditions. OTUD7B enhances calcium-dependent platelet activation and aggregation [47]. These regulatory effects indicate that DUB activity can influence both platelet responsiveness and thrombus formation. DUBs also modulate fibrinolysis through the regulation of key plasminogen-activation components. USP10 stabilizes tissue-type plasminogen activator (t-PA), thereby facilitating plasminogen activation and fibrin degradation. In contrast, OTULIN stabilizes plasminogen activator inhibitor-1 (PAI-1), which inhibits t-PA activity and suppresses fibrinolysis [47,48]. Therefore, the relative activity of DUBs acting on t-PA and PAI-1 may influence the balance between clot formation and thrombus resolution.

### 2.4. Functional Interplay Among DUBs: Redundancy, Complementarity, and Competition

**Figure 3:**
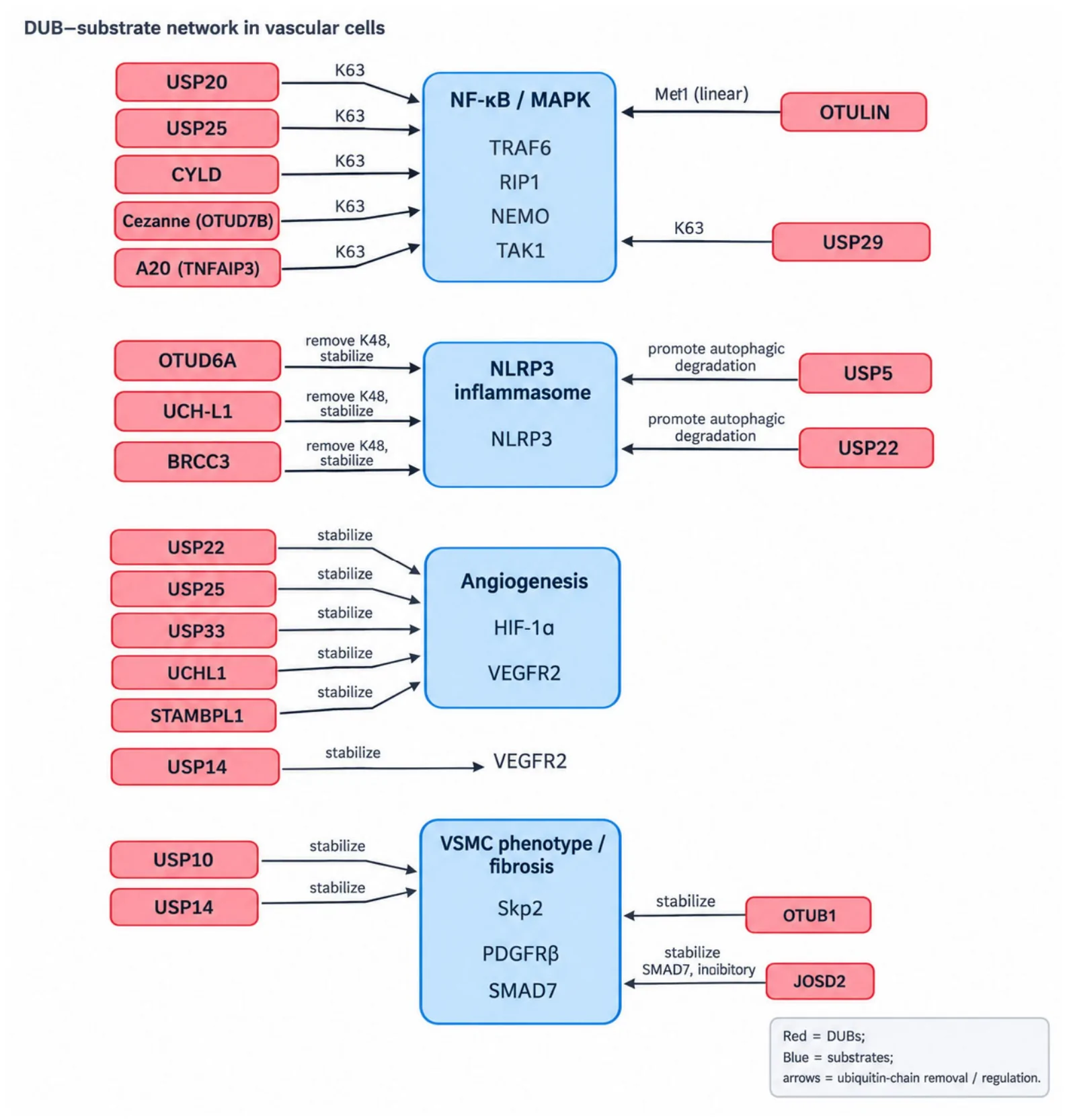
**Network of DUB–substrate interactions in vascular cells.** DUBs (red) converge on shared signaling nodes (blue) involved in inflammatory signaling (TRAF6, RIPK1, NEMO, TAK1, and NLRP3), vascular-cell phenotype and fibrotic signaling (Skp2, PDGFRβ, and SMAD7), angiogenesis (HIF-1α and VEGFR2), and hemostatic regulation. Multiple DUBs acting on a common signaling node illustrate functional redundancy, whereas selective removal of K63-, K48-, or Met1-linked ubiquitin chains illustrates linkage-specific complementarity. Opposing DUB activities toward NLRP3 exemplify competitive regulation of a shared substrate.

DUBs do not act as isolated enzyme–substrate pairs. Rather, their functions are integrated through functional redundancy, complementarity, and opposition, which collectively determine the outcome of ubiquitin-dependent signaling in vascular cells. First, functional redundancy occurs when several DUBs converge on shared signaling nodes. USP20, USP25, CYLD, Cezanne (OTUD7B), OTULIN, and A20 can all restrain NF-κB activation by editing ubiquitin chains on components of proximal signaling complexes, including TRAF6, RIPK1, and NEMO [3,4,5,6,7,8,9,10]. This overlapping regulation may provide buffering capacity, such that the loss or inhibition of a single DUB can be partially compensated for by other DUBs operating at the same or adjacent nodes. Second, DUBs display complementarity through linkage-specific and compartment-specific activities. OTULIN selectively removes Met1-linked linear ubiquitin chains generated by LUBAC, whereas USP20, USP25, CYLD, and Cezanne preferentially regulate K63-linked ubiquitin signals. In the NLRP3 pathway, OTUD6A, UCH-L1, and BRCC3 remove K48-linked ubiquitin chains and stabilize NLRP3 [3,4,5,6,7,8,9,10,14,15,16,17]. This division of labor enables DUBs to differentially regulate protein stability, localization, protein–protein interactions, and signaling output. Third, DUBs may exert opposing effects on the same signaling pathway. In the NLRP3 inflammasome pathway, USP5, USP22, and CYLD facilitate NLRP3 degradation through autophagy, whereas OTUD6A, UCH-L1, and BRCC3 preserve NLRP3 stability and promote inflammasome activation [14,15,16,17]. Consequently, the net effect of DUB activity depends on the relative expression, catalytic activity, substrate accessibility, ubiquitin-linkage specificity, and subcellular localization of multiple DUBs. Overall, DUB-mediated signaling in vascular cells is governed by an interconnected regulatory network rather than by individual enzyme–substrate interactions alone. This framework provides a mechanistic basis for the disease-specific and sometimes opposing functions of DUBs discussed in the following section (Figure 3).

## 3. Roles of Deubiquitinases in Vascular Diseases

The canonical signaling pathways described in Section 2 are engaged differently across vascular diseases. This section focuses on the disease-specific functions of DUBs, with particular emphasis on the effects of individual DUBs in distinct vascular beds, cell types, and pathological stages. Rather than reiterating the pathway-level mechanisms described above, we highlight how these mechanisms contribute to specific disease phenotypes and identify circumstances in which the same DUB exerts divergent or antagonistic effects.

### 3.1. Aortic Aneurysm

DUBs contribute to aortic aneurysm development through the coordinated regulation of VSMC survival, inflammation, oxidative stress, angiogenesis, and extracellular matrix remodeling. USP5 promotes aneurysmal progression by facilitating RIPK3-dependent VSMC necroptosis and by enhancing pathological angiogenesis through Notch-dependent STAT3 stabilization [49,50]. These findings suggest that USP5 may exert pathogenic effects through multiple cellular processes within the aneurysmal wall. USP10 further illustrates the context-dependent nature of DUB action in aneurysmal disease. In VSMCs, USP10 promotes inflammatory injury through the MKL1/NF-κB axis and may also influence proliferative responses through Skp2 stabilization [4,22,51]. CYLD promotes aneurysmal wall remodeling by stabilizing NOX4, thereby increasing oxidative stress and facilitating fibroblast-to-myofibroblast transdifferentiation. In addition, A20 exerts complex effects by limiting NF-κB-dependent inflammation while increasing VSMC susceptibility to apoptosis [52,53]. These findings indicate that the effects of a DUB cannot be inferred solely from its role in a single signaling pathway.

Preclinical studies demonstrate the therapeutic potential of targeting the ubiquitin system. Pharmacological strategies that enhance USP5-mediated necroptosis selectively in diseased VSMCs or broadly suppress ubiquitin activation (e.g., UBA1 inhibition) attenuate aneurysm progression [49]. Indirect suppression of OTUD6A by TEPP-46 attenuates aneurysm formation, consistent with the pathogenic role of NLRP3 stabilization in inflammatory vascular injury [16,54]. In addition, the ubiquitin-activating enzyme inhibitor TAK-243 suppresses aneurysm and dissection formation, accompanied by reductions in elastin fragmentation, extracellular matrix degradation, and macrophage infiltration [55]. However, because ubiquitin-system interventions can affect multiple cell populations and biological processes, the development of VSMC-, macrophage-, or disease stage-selective delivery approaches will be essential for clinical translation.

### 3.2. Atherosclerosis

DUBs exert multifaceted and often opposing roles in atherosclerosis by regulating inflammatory signaling, lipid handling, foam cell formation, and VSMC phenotypic switching. Atherosclerosis evolves over decades through endothelial activation, subendothelial retention of oxidized lipoproteins, monocyte recruitment and foam cell formation, VSMC migration and proliferation, and eventual plaque fibrosis or destabilization. The pro- and anti-atherogenic DUBs described below act at distinct points in this cascade, and their opposing functions reflect the cell type- and stage-specific logic of ubiquitin signaling in the arterial wall. Pro-atherogenic DUBs, including USP7, USP14, and BRCC3, promote lesion progression through stabilization of NF-κB activators, lipid uptake receptors, or NLRP3 inflammasome components [56]. In contrast, USP9X, USP18, USP20, and A20 limit disease development by suppressing inflammatory signaling or enhancing cholesterol efflux [5,10,18]. USP18 stabilizes ATP-binding cassette transporter G1 (ABCG1) by preventing its ubiquitination, thereby enhancing cholesterol efflux and limiting foam cell accumulation [57]. Conversely, USP14 promotes foam cell formation by stabilizing CD36, a major ox-LDL uptake receptor, while UCHL1 exerts an opposing effect by facilitating CD36 ubiquitination and proteasomal degradation [58,59]. Beyond endothelial and inflammatory cells, DUBs shape plaque evolution by acting on VSMCs: the Skp2/p27 proliferative axis introduced in Section 2.2 is actively engaged in the atherosclerotic wall, where USP10- and USP14-mediated Skp2 stabilization sustains the synthetic VSMC phenotype and promotes intimal hyperplasia [22,23]. Similarly, OTUB1-mediated stabilization of PDGFRβ amplifies PDGF-BB-driven VSMC dedifferentiation within developing lesions [24]. Conversely, JOSD2 opposes this program by stabilizing the TGF-β inhibitor SMAD7, restraining VSMC dedifferentiation in the arterial wall [26]. USP14 further contributes to lesion progression by stabilizing NLRC5, which activates SMAD2/3 signaling and promotes endothelial-to-mesenchymal transition and subsequent fibrosis [60]. The NLRP3-stabilizing DUB BRCC3 promotes atherogenesis by lowering the threshold for inflammasome activation and IL-1β production in the plaque [61].

Preclinical studies demonstrate that selective inhibition of pro-atherogenic DUBs, such as USP7 or BRCC3, attenuates inflammation, fibrosis, and plaque progression [61,62]. Additionally, Panax notoginseng saponins exert anti-atherosclerotic effects by inhibiting USP2-mediated deubiquitination of Keap1, which stabilizes Nrf2 and activates antioxidant and ferroptosis-inhibitory pathways [63]. These findings highlight DUBs as attractive therapeutic targets, while emphasizing the need for high selectivity to avoid disrupting protective DUB functions.

### 3.3. Pulmonary Hypertension

DUB dysregulation contributes to pulmonary arterial hypertension (PAH) primarily through effects on pulmonary artery smooth muscle cell (PASMC) proliferation, apoptosis resistance, metabolic adaptation, and pulmonary vascular remodeling. Ubiquitin carboxyl-terminal hydrolase L1 (UCHL1) drives PAH by stabilizing AKT and other pro-proliferative, pro-survival proteins, promoting pulmonary artery smooth muscle cell (PASMC) proliferation, apoptosis resistance, and metabolic reprogramming [64]. Pharmacological inhibition of UCHL1 with LDN-57444 restores ubiquitin-dependent AKT degradation, reversing vascular remodeling, reducing pulmonary pressure, and normalizing metabolism in preclinical models. Similarly, irisin recruits NEDD4 to degrade ENO1, suppressing PASMC proliferation and attenuating remodeling [64,65]. Targeted DUB inhibition therefore represents a mechanistically distinct therapeutic approach. However, clinical translation will require lung-specific delivery to minimize off-target effects, optimal timing during active remodeling, and combination with BMPR2 agonists, prostacyclin analogs, endothelin receptor antagonists, or activin inhibitors for synergistic disease modification. Future work should focus on selective pulmonary targeting, defining therapeutic windows, and establishing combination regimens to fully leverage DUB inhibition for disease modification.

### 3.4. Ischemic Stroke

In ischemic stroke, DUBs regulate neuronal survival, mitochondrial quality control, neuroinflammation, programmed cell death, and post-ischemic synaptic remodeling. In contrast to the pro-inflammatory or pro-remodeling effects of certain DUBs in peripheral vascular disorders, several DUBs are protective in the ischemic brain. Neuroprotective DUBs include USP7, which stabilizes SIRT1 to promote PINK1/Parkin-mediated mitophagy and mitochondrial biogenesis [66]. USP22 stabilizes β-catenin within transcriptional complexes, facilitating synapse formation and neuroplasticity, particularly during hypoxia postconditioning [67]. By contrast, USP15 is detrimental in ischemic stroke, promoting neuronal disulfidptosis through deubiquitination of SETD1B; accordingly, its inhibition with chaetocin confers neuroprotection [68]. In the ischemic brain, USP25 dampens neuroinflammation through the TAB2-directed mechanism, restraining microglial activation and NF-κB signaling after stroke [69]. OTUD1, which stabilizes PINK1 to promote mitophagy, supports neuronal survival in the ischemic brain, whereas OTUD3 accelerates apoptosis by stabilizing caspase-3 [70]. OTUB2 exacerbates necroptotic cell death by preventing proteasomal degradation of RIPK3, thereby facilitating necrosome assembly and MLKL-mediated plasma membrane rupture [71].

Preclinical interventions targeting these DUBs, such as Alisol B (enhancing USP7), chaetocin (inhibiting USP15-SETD1B), OTUB2 inhibitors, or exosome-mediated USP25 delivery, demonstrate substantial neuroprotection [66,68,69,70,71].

Despite these promising findings, clinical translation remains challenging due to cell type- and stage-specific DUB functions, compensatory upregulation of related DUBs, and systemic off-target effects. Brain-targeted delivery systems, including BBB-penetrating nanoparticles, and combination strategies targeting multiple DUBs within functional networks, will be essential to harness DUB modulation as an effective stroke therapy.

### 3.5. Thrombotic Diseases and Thrombophilia

DUBs regulate thrombotic disease through coordinated effects on endothelial activation, platelet reactivity, coagulation, and fibrinolysis. USP7 and USP14 promote endothelial prothrombotic transformation and ferroptosis via TLR4/NF-κB and p53/SLC7A11/GPX4 pathways, while CYLD counteracts excessive NF-κB signaling, highlighting context-dependent effects [48,72]. In platelets, USP9X stabilizes the GPIb-IX complex to support adhesion, whereas OTUD7B enhances calcium-mediated aggregation, particularly in diabetes [47]. DUBs also modulate fibrinolysis: USP10 stabilizes t-PA to accelerate thrombolysis, whereas OTULIN stabilizes PAI-1, favoring thrombus persistence [47,48]. These findings suggest that the impact of DUB modulation on thrombosis depends on the balance between endothelial protection and inflammatory activation.

Preclinical interventions—such as CYLD activation via dCas9-SAM or OTUD7B inhibition with XY-421—effectively reduce pathological thrombosis without impairing hemostasis. These findings underscore the therapeutic potential of DUB-targeted precision antithrombotics. Future translation will require selective modulators, cell- and vessel-targeted delivery systems, and clinical validation in high-risk populations. By leveraging the substrate specificity and context dependence of DUBs, next-generation therapies may suppress pathological thrombosis while minimizing bleeding risk [47,48].

### 3.6. Deubiquitinases in Intimal Hyperplasia and In-Stent Restenosis

Deubiquitinases critically regulate intimal hyperplasia and restenosis by modulating VSMC phenotypic switching, proliferation, and survival. The Skp2/p27 proliferative axis is a central driver of post-injury neointima formation: USP10-mediated Skp2 stabilization promotes VSMC G1/S progression and neointimal hyperplasia, whereas USP18 and USP48 exert protective effects by restraining JAK-STAT/NF-κB signaling and stabilizing p53, respectively [22,73]. Preclinical studies demonstrate that USP10 inhibition, via small molecules or RNA interference, restores ubiquitin-dependent degradation of Skp2, arrests VSMC proliferation, and markedly reduces neointimal hyperplasia. Combined approaches that enhance endothelial repair, such as M2 macrophage-derived exosomes, further mitigate restenosis, highlighting the therapeutic potential of dual targeting [74].

Despite these promising results, clinical translation faces challenges due to temporal and context-dependent DUB functions and ubiquitous expression. Strategies for vascular-specific, stage-restricted modulation—such as stent-based drug delivery, endothelial-targeted nanoparticles, or injury-responsive expression systems—will be critical to maximize efficacy while minimizing off-target effects. Targeted DUB modulation thus represents a mechanistically distinct and highly promising approach to prevent restenosis by balancing VSMC proliferation and vascular repair.

## 4. Therapeutic Strategies and Translational Status of Targeting DUBs

### 4.1. Small-Molecule Inhibitors

**Figure 4:**
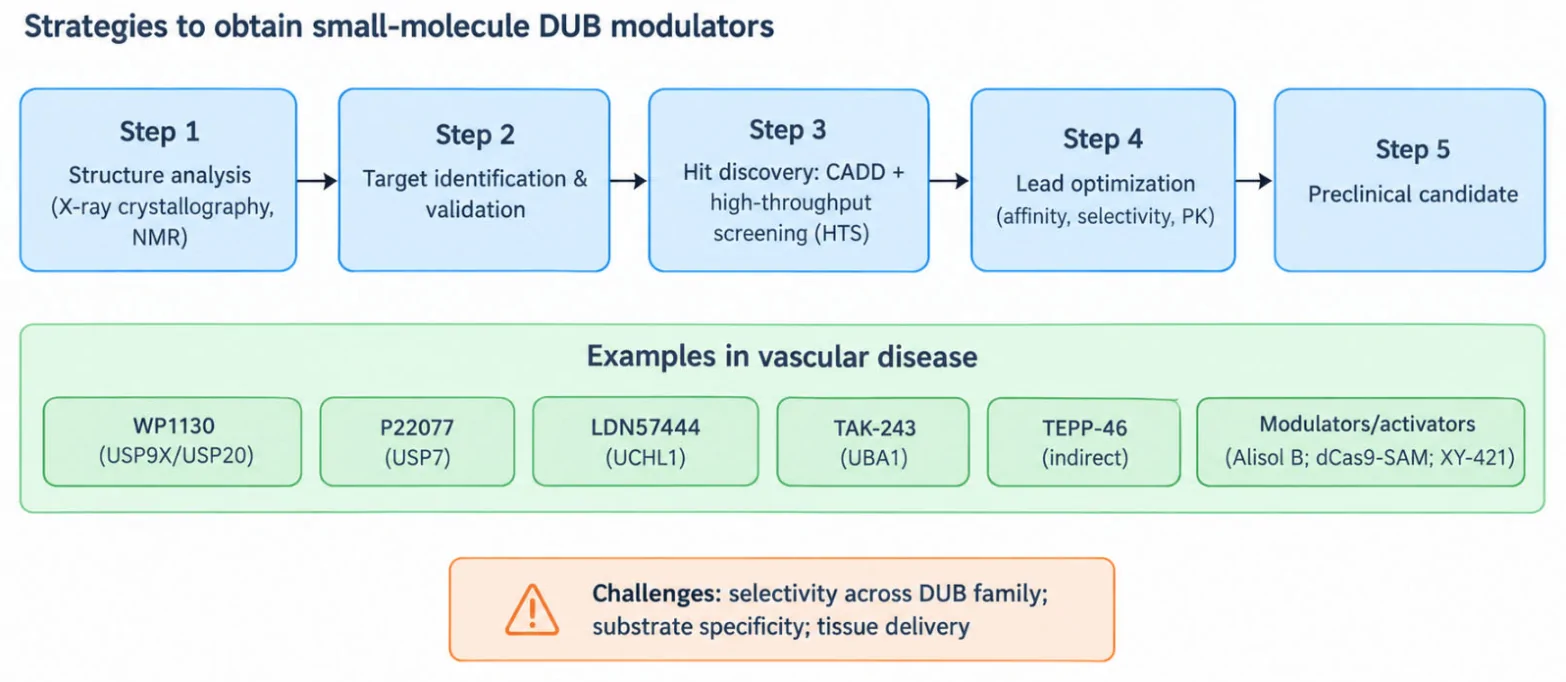
**Strategies for developing small-molecule modulators of DUB activity.** Structure-based approaches (X-ray crystallography, NMR) define catalytic and substrate-binding pockets; computer-aided drug design (CADD) and high-throughput screening (HTS) identify candidate scaffolds that are subsequently optimized for affinity, selectivity, and pharmacokinetic properties. Examples discussed in this review include WP1130, P22077, LDN57444, TAK-243, TEPP-46, and activators/modalities such as Alisol B, dCas9-SAM, and XY-421. Key challenges include selectivity across structurally related DUBs, substrate specificity, and tissue delivery.

Given the central roles of deubiquitinases (DUBs) in vascular pathophysiology, small-molecule inhibitors have emerged as a promising therapeutic approach. Development strategies rely on detailed structural and functional analyses. Techniques such as X-ray crystallography and NMR spectroscopy reveal the three-dimensional configuration of DUBs, including substrate-binding pockets, which inform rational drug design. Computer-aided drug design (CADD) and high-throughput screening (HTS) enable virtual and experimental identification of candidate compounds, followed by chemical optimization to enhance affinity, selectivity, and pharmacokinetic properties [75,76,77,78,79] (Figure 4).

Preclinical studies demonstrate that DUB inhibition can modulate disease-relevant pathways. For example, WP1130 inhibits USP20 and USP9X in macrophages, suppressing pro-inflammatory cytokine expression and ox-LDL uptake, thereby attenuating atherosclerotic plaque formation. In pulmonary arterial hypertension models, LDN57444, a selective UCHL1 inhibitor, reduces endothelial and smooth muscle cell proliferation, decreases right ventricular hypertrophy, and reverses vascular remodeling. These findings highlight the therapeutic potential of targeting DUBs to restore ubiquitin-dependent protein turnover and normalize pathological signaling [18,64,80].

### 4.2. Gene Therapy

#### 4.2.1. Viral Vector

Adeno-associated virus (AAV) and lentiviral vectors are widely employed for DUB-targeted gene therapy due to their high delivery efficiency. AAV vectors enable long-term expression with low immunogenicity, suitable for silencing or overexpressing DUBs in specific cell populations. For instance, AAV-mediated USP18 overexpression protects neurons in spinal cord ischemia–reperfusion models by inhibiting apoptosis, neuroinflammation, and dysregulated autophagy. Lentiviral vectors allow stable integration into dividing and non-dividing cells and have been used to deliver USP9X in hindlimb ischemia models, improving perfusion and promoting angiogenesis [10,73,81].

#### 4.2.2. Non-Viral Vector Delivery

Non-viral platforms, including liposomes and polymeric nanoparticles, offer safer alternatives. Liposomes encapsulate nucleic acids (siRNA or plasmids) via electrostatic or hydrophobic interactions, while nanoparticles (e.g., PLGA-based) allow surface functionalization with targeting ligands for vascular-specific delivery. These systems have effectively delivered DUB-targeting siRNAs, such as USP44, to VSMCs in vitro and in vivo, suppressing proliferation and reducing intimal hyperplasia [73].

#### 4.2.3. Gene Editing Technologies for Targeting DUBs

CRISPR-Cas9 offers precise manipulation of DUB genes by inducing targeted DNA cleavage and repair. This technology enables correction of pathogenic mutations or knockout of pro-disease DUBs. However, clinical application is limited by off-target cleavage, delivery challenges, and potential immunogenicity. Emerging strategies, including high-fidelity Cas9 variants and nanoparticle-mediated delivery, aim to enhance specificity and efficiency [73].

### 4.3. Difficulties and Challenges in Clinical Translation

#### 4.3.1. Safety Concerns: Potential Off-Target Effects

Off-target effects pose a significant safety risk in the clinical translation of small-molecule inhibitors. Due to structural similarities among DUB family members, inhibitors may bind non-specifically to non-target DUBs or other proteins. For example, WP1130 is the most well-known USP9X inhibitor and is reported to affect other DUBs (USP5, USP14, and UCHL5) [82,83].

Gene therapies also carry off-target risks. Both viral and non-viral delivery systems may result in transgene expression in unintended cells [73]. For instance, although engineered AAV vectors can be designed to target specific cells, they may still infect off-target cells in the complex in vivo environment. If an AAV vector carrying USP33 shRNA inadvertently infects germ cells, foreign gene integration into the germline genome could have potential hereditary implications. Non-viral vectors face similar issues; liposomes or nanoparticles circulating in the blood may be taken up by non-target cells, leading to misdelivery and expression. Moreover, the off-target effects of gene editing technologies are even more pronounced. Off-target cleavage by CRISPR-Cas9 may cause unintended mutations, increase the risk of carcinogenesis and pose serious threats to patient health, thereby hindering clinical translation.

#### 4.3.2. Validation of Efficacy

At the preclinical stage, differences between animal models and human vascular diseases make it difficult to extrapolate the efficacy of therapeutic strategies to humans. Vascular anatomy, physiological functions, and disease mechanisms in animals are not identical to those in humans. For example, although mouse models of atherosclerosis mimic some features of the human disease, there are significant differences in lipid metabolism and inflammatory regulation [3]. A treatment strategy targeting USP20 that is effective in mice may show limited efficacy in human clinical trials. Furthermore, accurately assessing the efficacy of DUB-targeted therapies in human clinical trials is challenging. Vascular diseases are complex, and a single biomarker often cannot fully reflect therapeutic outcomes. Current evaluation methods—such as angiography and blood biochemical indicators—cannot precisely measure the profound effects of DUB-targeted therapies on vascular cell function and the vascular microenvironment. Therefore, there is a need to develop more comprehensive and precise biomarkers and detection technologies to accurately evaluate the efficacy of these therapies in humans and provide a reliable basis for clinical decision-making [73].

#### 4.3.3. Cost and Accessibility

The development and production of small-molecule inhibitors are costly, limiting their clinical application. Each stage-from structure-based drug design and high-throughput screening to lead optimization and clinical trials-requires substantial financial investment. Moreover, the large-scale production of small-molecule inhibitors involves complex processes and strict quality control, further increasing costs. For example, WP1130 is a research-grade DUB inhibitor whose synthesis requires specialized techniques and stringent quality control, and its development reflects the substantial cost associated with structure-based design, screening, and lead optimization that generally limits the clinical accessibility of small-molecule DUB inhibitors. Its production requires specialized synthesis techniques and expensive raw materials, resulting in a high market price that is unaffordable for many patients, greatly restricting its clinical use.

Gene therapy regimens also face cost challenges. The production of viral vectors is complex, requiring specialized cell culture facilities, strict sterile operating environments, and high-purity raw materials, all of which contribute to high production costs. Although non-viral vectors are relatively less expensive, achieving desirable gene delivery often requires multiple administrations or large vector quantities, leading to considerable overall costs. Additionally, gene therapy requires specialized medical teams for operation and monitoring, and healthcare institutions must be equipped with advanced genetic testing and treatment equipment. These factors increase treatment costs, making gene therapy less accessible and difficult to widely implement for the benefit of patients [73].

## 5. Conclusions and Future Directions

**Figure 5:**
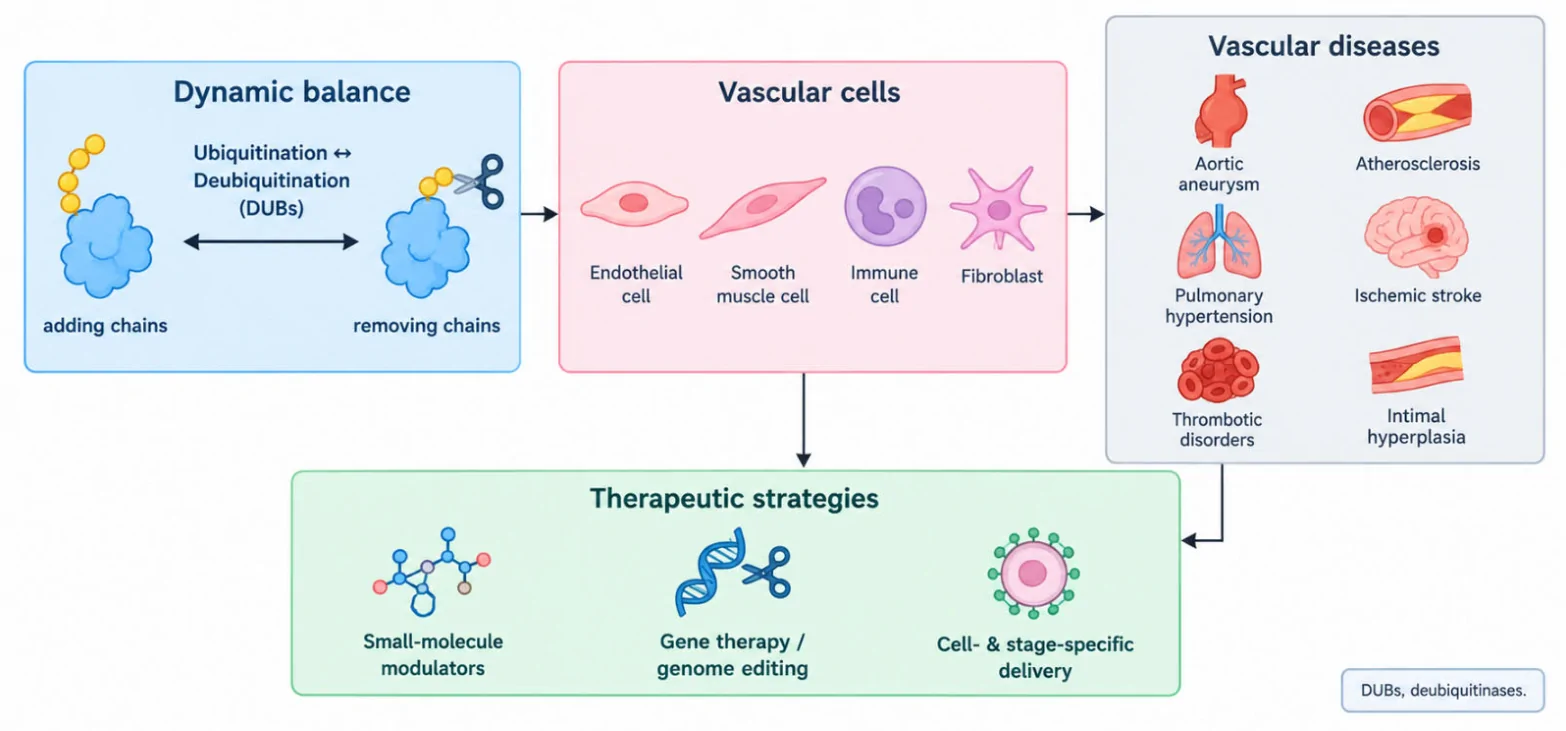
**Summary of deubiquitinase function in vascular disease and therapeutic outlook.** DUBs maintain the dynamic balance between ubiquitination and deubiquitination across vascular cell types (endothelial cells, smooth muscle cells, immune cells, and fibroblasts). Deregulation of distinct DUB–substrate axes contributes to vascular inflammation, remodeling, and thrombosis, and thereby to aortic aneurysm, atherosclerosis, pulmonary hypertension, ischemic stroke, thrombotic disorders, and intimal hyperplasia. Therapeutic modulation of DUBs—by small molecules, gene-based strategies, or cell- and stage-specific delivery—holds promise but requires context-dependent targeting.

Deubiquitinases act as central regulators of vascular homeostasis by controlling the stability and activity of key signaling proteins. Dysregulation of DUBs contributes to a spectrum of vascular diseases, including atherosclerosis, pulmonary hypertension, stroke, thrombosis, and restenosis, in a cell type- and context-specific manner. Preclinical evidence highlights the therapeutic potential of targeting DUBs with small molecules, gene therapy, and genome-editing technologies. Future research should focus on: (1) comprehensive mapping of DUB–substrate networks within specific vascular cell types and microenvironments; (2) development of highly selective inhibitors and tissue-targeted delivery systems to mitigate off-target effects; and (3) elucidation of interactions between deubiquitination and other post-translational modifications. Targeted modulation of DUB activity represents a mechanistically distinct and promising strategy to restore protein homeostasis, curb pathological vascular remodeling, and improve patient outcomes in diverse cardiovascular diseases (Figure 5).

## Data Availability

No new data were created or analyzed in this study. Data sharing is not applicable to this article.
